# PLAU1 Facilitated Proliferation, Invasion, and Metastasis *via* Interaction With MMP1 in Head and Neck Squamous Carcinoma

**DOI:** 10.3389/fonc.2021.574260

**Published:** 2021-03-18

**Authors:** Kun Wu, Yuan-Yuan Mao, Nan-Nan Han, Hanjiang Wu, Sheng Zhang

**Affiliations:** ^1^ Department of Stomatology, Second Xiangya Hospital, Central South University, Changsha, China; ^2^ Department of Oral Maxillofacial-Head and Neck Oncology, Shanghai Ninth People’s Hospital, School of Medicine, Shanghai Jiao Tong University, Shanghai, China

**Keywords:** MMP1, metastasis, invasion, head and neck squamous cell carcinoma, PLAU1

## Abstract

Head and neck squamous cell carcinoma (HNSCC) is the sixth most common malignant neoplasm; it is associated with high morbidity and mortality. Thus, understanding the molecular mechanisms underlying its initiation and progression is critical for establishing the most appropriate treatment strategies. We found that urokinase-type plasminogen activator (PLAU1) was upregulated and associated with poor prognosis in HNSCC. Silencing of PLAU1 inhibited the proliferation, colony-formation, migration, and invasion abilities of HNSCC cells *in vitro* and reduced the expression of matrix metalloproteinase 1 (MMP1), whereas PLAU1 overexpression significantly enhanced the growth, the colony-formation, migration, and invasion abilities, and the xenograft tumor growth of HNSCC cells *in vivo* and increased the expression of MMP1. The Co-IP assay verified that PLAU1 interacted with MMP1. A positive correlation between PLAU1 and MMP1 expression was observed in HNSCC samples. si-RNAs against MMP1 reversed the aggressive effects of PLAU1 overexpression in HNSCC. Taken together, our data revealed that PLAU1 facilitated HNSCC cell proliferation, invasion, and metastasis *via* interaction with MMP1.

## Introduction

Head and neck squamous cell carcinoma (HNSCC) is the sixth most common malignant neoplasm; it is associated with high morbidity and mortality (1, 2), with approximately 800,000 new HNSCC cases reported annually worldwide (3). Despite therapeutic advancements in the last 40 years, the 5-year overall survival of HNSCC patients is still about 50% (4). Malignant tumor recurrence and metastasis continues to be responsible for the adverse effects of HNSCC treatments, which has led to poor outcomes, including the occurrence of relapses (5). Hence, a better understanding of the molecular mechanisms underlying HNSCC initiation and progression is critical for establishing the most appropriate treatment strategies for this condition.

Urokinase-type plasminogen activator (PLAU1), which is overexpressed in different human cancer types (6–9), encodes a secreted serine protease that converts plasminogen to plasmin; this enzyme can promote tumor cell invasion and metastasis by degrading the components surrounding the extracellular matrix (10, 11). PLAU1 also acts as a biomarker to predict prognosis in breast cancer and non-small-cell lung cancer (12, 13); synthetic antibodies against PLAU1 have been used to inhibit cancer progression (14). Moreover, the level of PLAU1 was used for the risk assessment of node-negative breast cancer, according to the guidelines of the American Society of Clinical Oncology (15). Furthermore, the biological role and molecular mechanisms underlying the action of PLAU1 in HNSCC cells have not yet been elucidated.

In the present study, the biological function and expression levels of PLAU1 were evaluated by qRT-PCR, loss-of-function, and gain-of-function assays *in vitro* and *in vivo*. The molecular mechanisms whereby PLAU1 facilitated HNSCC progression were determined by correlation analysis, co-IP assays, and rescue assays *in vitro*.

## Material and Methods

### Patients and Specimens

Eighty pairs of tumor and adjacent normal tissues were collected from primary HNSCC patients who underwent an initial surgical treatment between June 2017 and March 2019 and presented to the Department of Oral and Maxillofacial Surgery at the Second Xiangya Hospital of Central South University. These HNSCC patients did not show distant metastases and had not received any systemic treatment before primary surgery. Patients with a history of malignancy were excluded. Tumor specimens from these patients were quickly frozen in liquid nitrogen until the total RNA was extracted. Clinical data were obtained from medical records. The tumors were diagnosed and staged according to the 8th edition of the UICC cancer staging manual. This study was approved by the Ethics Committee of the Second Xiangya Hospital, and written informed consent was obtained from all participants.

### Cell Culture and Reagents

HN30 cells were kindly provided by the University of Maryland Dental School, USA. Cal27 cells were purchased from the American Type Culture Collection (MD, USA). HN30 and Cal27 cells were obtained from primary pharynx squamous carcinoma and tongue squamous carcinoma, respectively. The cells were cultured in Dulbecco’s modified Eagle’s medium (Gibco-BRL, CA, USA) containing 10% fetal bovine serum (Gibco-BRL, CA, USA), streptomycin (100 μg/mL), and penicillin (100 units/mL) at 37°C in a humidified 5% CO_2_ atmosphere.

### RNA Extraction and Real-Time PCR Analysis

Total RNA was extracted from the tumor tissues and cells using TRIzol reagent (Takara, Japan), and reverse-transcribed into cDNA using a PrimeScript RT Reagent Kit (Takara, Japan). Real-time PCR (RT-PCR) was performed at a final reaction volume of 20 μL with 1 μL of template cDNA at a concentration of 20 ng/μL, using the SYBR Premix Ex Taq Reagent Kit (Takara, Japan) on the StepOne RT-PCR System (Life Technologies, USA), according to the manufacturer’s instructions. The mRNA levels were normalized to β-actin levels. The primer sequences used for RT-PCR in the present study are listed in **Supplementary Table 2**.

### Cell Transfection

The siRNAs against PLAU1 and matrix metalloproteinase 1 (MMP1) (si-PLAU1, si-MMP1) and si-Scramble (si-NC) (Ribobio, China) were transfected into HNSCC cells using Lipofectamine RNAiMAX (Invitrogen, USA) at a final concentration of 100 nM. The transfected cells were harvested, and the corresponding experiments were performed after a 24-h incubation.

### Lentiviral Transduction and Screening of Stable Strains

Transduction of the cells with PLAU1 lentiviral expression vectors (LV-PLAU1) and NC (LV-NC), conferring puromycin resistance, were performed by HanYin Biotechnology. Lentiviral transduction was performed according to the manufacturer’s instructions. After 72 h, the cells were cultured in medium containing puromycin at a final concentration of 3–10 mg/mL. After being passaged two to three times, stably stained cells were screened.

### Western Blotting Analysis

The total protein of HNSCC cells was extracted using SEMS lysis buffer (Beyotime, China); the protein contents were determined using a BCA protein assay kit (Beyotime, China). Equal amounts of proteins (40 μg) were separated using premade SDS-PAGE gels (Jinsirui, China) and transferred onto 0.22-μm polyvinylidene fluoride membranes (Merck Millipore, USA). The membranes were then incubated in 10% skim milk in PBS for 1 h at room temperature and incubated with the primary antibodies (1:1000) at 4°C overnight. The membranes were then probed with HRP-labeled rabbit antibodies (Beyotime, China) for 1 h, and an ECL (Beyotime, China) developing solution was added to enable the visualization of the protein bands, which were detected on a chemiluminometer (Amersham Imager 600). The antibodies against PLAU1, MMP1, MMP7, TIMP2, CDH1, and GAPDH were purchased from Abcam (UK).

### MTT Assay

The cells stably transduced with the lentiviral constructs or those transfected for 24 h with the siRNAs were seeded into 96-well plates at a density of 1,000 cells per well with 100 μL of medium at 37°C. The cells were incubated with 3-(4,5-dimethylthiazol-2-yl)-2,5-diphenyltetrazolium bromide (MTT) (Sigma-Aldrich, USA) at a final concentration of 0.5 mg/mL in the culture medium for 4 h. The resulting formazan was solubilized in 150 μL of dimethyl sulfoxide, and the absorbance of the samples was measured using a multi-well plate reader (Bio-Rad Laboratories, Hercules, CA, USA) at 490 nm.

### Colony-Formation Assay

Cells were seeded into 6-well plates with 1000 cells per well and incubated for 10–14 days until obvious clonal proliferation was observed. The colonies were fixed with paraformaldehyde and stained with 0.1% crystal violet for 1 h. The images were recorded using a scanner (CanoScan 5600F), which quantified the number of clones containing more than 50 cells.

### Wound-Healing Assay

Cells were seeded in 6-well plates and incubated until confluence. Then, the cells were scraped with a P200 tip (0 h), washed with PBS, and incubated with serum-free Dulbecco’s Modified Eagle Medium (DMEM). Five nonoverlapping field images were collected at 24 h.

### Transwell Invasion Assay

Cell invasion assays were performed using the Transwell chamber (pore size, 0.8 μm; Merck Millipore) coated with Matrigel (BD Biosciences) at a cell density of 3 × 10^5^ cells/well. The cell suspension in serum-free medium was added into each upper chamber, while DMEM containing 20% FBS was added to the lower chambers as a chemo-attractant. After incubation for 24–36 h, invaded cells were fixed and stained with 0.1% crystal violet for 1 h, and then washed with PBS. The images of five fields were randomly selected, and the cells were counted.

### Pulmonary Metastasis Assay *In Vivo*

All animal experiments, performed using four-week-old BALB/C nude mice (Shanghai Laboratory Animal Center), were performed in accordance with the national guidelines and ethical standards. Cal27 cells (1×10^6^) stably overexpressing LV- PLAU1 or LV-NC were injected into the tail vein (n = 6 in each group). After day 36, the mice were sacrificed, and the lung samples were harvested. Metastatic nodules on the mouse lung surfaces were detected by neutral aldehyde and picric acid staining.

### Immunohistochemical Analysis

Briefly, paraffin-embedded tissue sections were deparaffinized, rehydrated, and immersed in EDTA buffer for heat-induced antigen retrieval. Then, the sections were immersed in 0.3% hydrogen peroxide to block endogenous peroxidase activity, blocked with 10% goat serum albumin, and incubated with MMP1 primary antibodies at 4°C overnight; then, they were developed using the DAKO ChemMate Envision Kit/HRP (Dako-Cytomation, USA), according to the manufacturer’s instructions. Finally, the sections were counterstained with hematoxylin, dehydrated, cleared, and mounted.

### Co-immunoprecipitation (Co-IP) Analysis

Co-immunoprecipitation (Co-IP) analysis was performed as described in our previous study (16). Briefly, the cells were lysed with IP buffer (proteins at a concentration of 1 mg/mL) and incubated with antibodies against PLAU1 (1:20, Proteintech, China) or MMP1 (1:20, Abcam, UK) at 4°C overnight, followed by incubation with protein A/G Magbeads (Merck) at 4°C for 6 h. The proteins were then separated from the Magbeads by treatment at 105°C for 10 min. The supernatants were then obtained for subsequent immunoblotting analyses.

### Statistical Analysis

The Kruskal-Wallis tests and Mann-Whitney U tests were used to assess the association between the PLAU1 and MMP1 levels and clinical parameters. The significance between two or more groups was determined selectively by one-way ANOVA or Student’s t-test. Pearson correlation analysis was used to analyze the correlation between two variables. The HNSCC patients were divided into the low-and high-level groups according to the median PLAU1 levels, and Kaplan–Meier survival analysis was used to compare HNSCC patient survival based on dichotomized gene expression using the log-rank test. The receiver operating characteristic (ROC) curve was used to discriminate tumor tissues from the adjacent normal tissues. The data were analyzed using SPSS 19.0 (SPSS, Chicago, IL, USA). All values were two-sided, and differences with p values < 0.05 were considered statistically significant.

## Results

### PLAU1 Was Upregulated and Associated With Poor Prognosis in HNSCC

**Figure 1:**
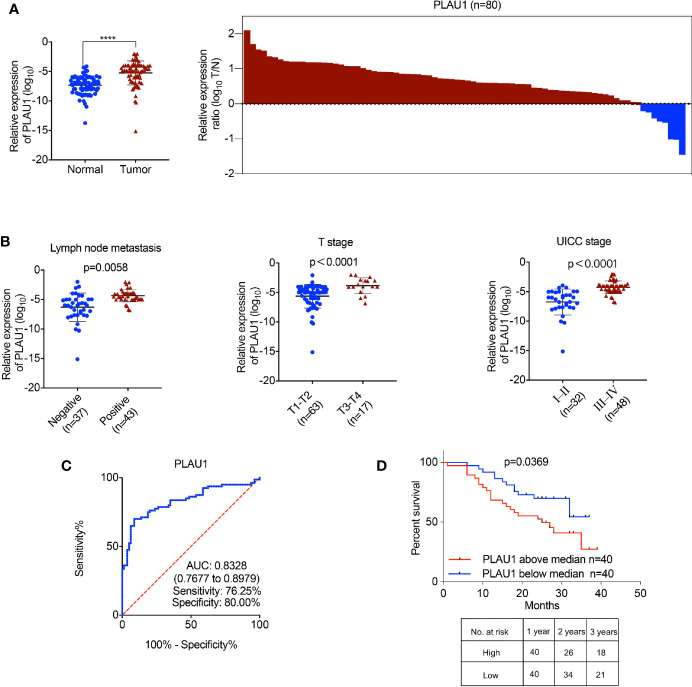
The expression of PLAU1 in HNSCC tissues. **(A)** The PLAU1 expression level was significantly upregulated in HNSCC tissues, compared to that in paired adjacent normal tissues (****p < 0.0001). **(B)** Upregulated PLAU1 levels were correlated with increased tumor size, lymph node metastasis, and advanced tumor stage. **(C)** ROC curves of PLAU1 for discriminating HNSCC tissues from adjacent normal tissues. **(D)** Kaplan-Meier analysis of overall survival. Compared with patients with low PLAU1 expression, those with high PLAU1 expression had a significantly lower overall survival rate. ROC: receiver operating characteristic curve.

To identify the PLAU1 expression levels in tumor specimens of patients with HNSCC, PLAU1 expression was examined by RT-PCR in 80 samples of tumor tissues paired with adjacent normal tissues. PLAU1 expression levels were significantly elevated in HNSCC tissues (**Figure 1A**). Further analysis revealed that PLAU1 was correlated with metastatic lymph nodes, T stage, and UICC stage (**Figure 1B**). In addition, the area under curve (AUC) of PLAU1 was 0.8328 (95% CI, 0.7677–0.8979; sensitivity: 76.25%; specificity: 80.00%) (**Figure 1C**). High levels of PLAU1 were associated with poor outcomes in HNSCC patients (**Figure 1D**). An analysis of The Cancer Genome Atlas (TCGA) database further revealed that PLAU1 was also significantly upregulated in HNSCC samples (**Supplementary Figure 1A**), and high levels of PLAU1 were associated with poor outcomes (**Supplementary Figure 1B**). These findings showed that PLAU1 is upregulated in HNSCC tissues and may promote HNSCC progression.

### Knockdown of PLAU1 Suppressed HNSCC Cell Proliferation, Migration, and Invasion *In Vitro*

**Figure 2:**
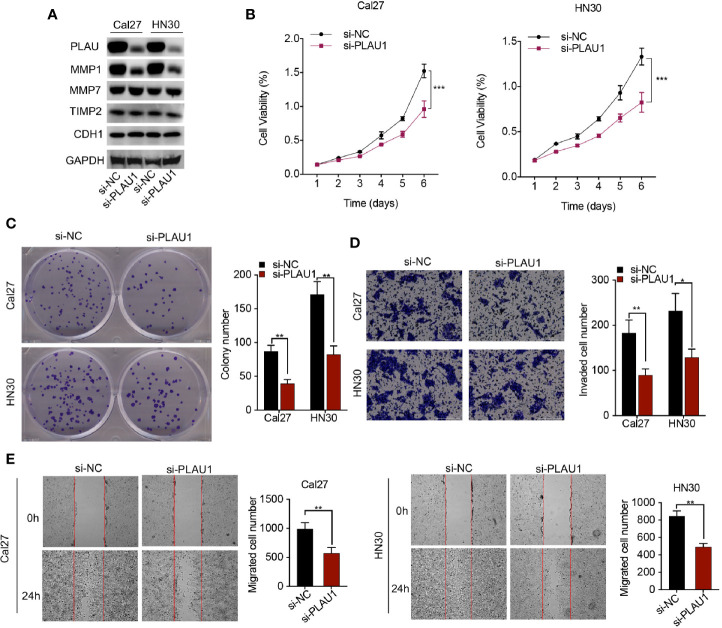
PLAU1 knockdown inhibited HNSCC cell proliferation, migration, and invasion *in vitro*. **(A)** The expression levels of PLAU1, MMP1, MMP7, TIMP2, and CDH1 were detected by western blotting in HNSCC cells showing PLAU1 downregulation. **(B–E)** The growth curve and colony-formation, invasion, and migration abilities of HNSCC cells with PLAU1 knockdown were detected using MTT assays **(B)**, colony-formation assays **(C)**, Transwell assays **(D)**, and wound-healing assays **(E)**, respectively. Data are presented as the mean ± SD from three independent experiments (*p < 0.05, **p < 0.01, ***p < 0.001). HNSCC: Head and neck squamous cell carcinoma.

To evaluate the biological function of PLAU1 knockdown in cells, siRNAs against PLAU1 were synthesized (5’-ATGAATGTATCAGGAAATATATA-3’), and the silencing effect of siRNAs against PLAU1 was detected by western blotting assays in Cal27 and HN30 cells. Upon PLAU1 knockdown, the post-translational level of PLAU1 was markedly decreased in HNSCC cells (**Figure 2A**). Additionally, the expression level of the MMP1 protein was correspondingly reduced following PLAU1 knockdown. However, there were no significant alterations in the expression levels of the MMP7, TIMP2, and CDH1 proteins following PLAU1 silencing (**Figure 2A**). Knockdown of PLAU1 decreased the proliferation and colony-formation abilities of HN30 and Cal27 cells, compared to the cells in the control group (**Figures 2B, C**). Furthermore, the invasion and migration abilities of HN30 and Cal27 cells with PLAU1 knockdown were inhibited, compared to those of the cells from the control group, as assessed by the wound-healing and Transwell assays (**Figures 2D, E**). To exclude the unspecific effects, the untreated cells were included in transfection experiments (**Supplementary Figure 2**). However, the downregulation of PLAU1 also reduced cell proliferation, which may affect the reduction of the invasion and migration abilities of the cells, due to the presence of a fewer number of cells.

### PLAU1 Overexpression Promoted HNSCC Cell Proliferation, Migration, and Invasion *In Vitro*

**Figure 3:**
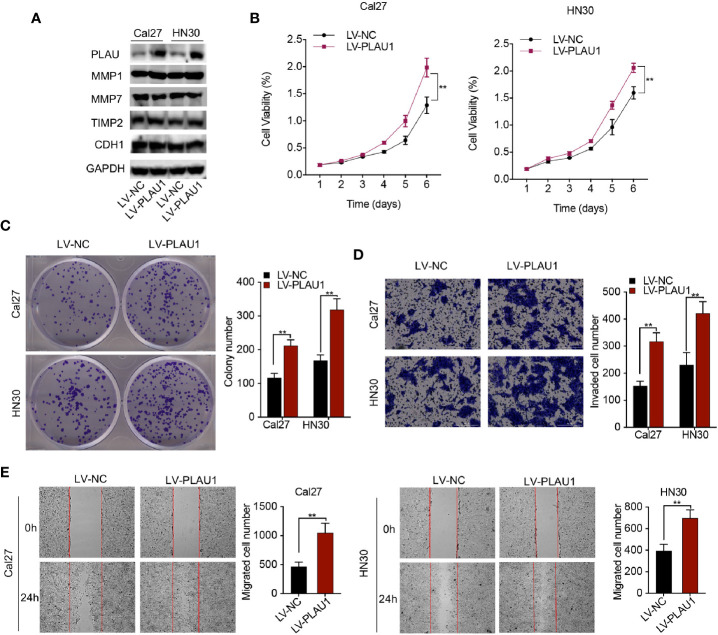
PLAU1 overexpression promoted HNSCC cell proliferation, migration, invasion *in vitro*. **(A)** The expression levels of PLAU1, MMP1, MMP7, TIMP2, and CDH1 were detected by western blotting in HNSCC cells with PLAU1 overexpression. (B–E) The growth curve and colony-formation, invasion, and migration abilities of HNSCC cells with PLAU1 overexpression were detected using MTT assays **(B)**, colony-formation assays **(C)**, Transwell assays **(D)**, and wound-healing assays **(E)**, respectively. Data are presented as the mean ± SD from three independent experiments (**p < 0.01).

To investigate the biological function of PLAU1 overexpression in cells, PLAU1 levels were notably increased after lentivirus infection (**Figure 3A**). Additionally, the expression level of MMP1 protein increased following PLAU1 overexpression. However, there were no significant alterations in the expression levels of the MMP7, TIMP2, and CDH1 proteins after PLAU1 overexpression (**Figure 3A**). Overexpression of PLAU1 significantly facilitated cell proliferation (**Figure 3B**), and enhanced the colony-formation, invasion, and migration abilities of the cells (**Figures 3C–E**). However, enhanced proliferation can also lead to a higher number of invading/migrating cells.

### PLAU1 Interacted With MMP1 in HNSCC

**Figure 4:**
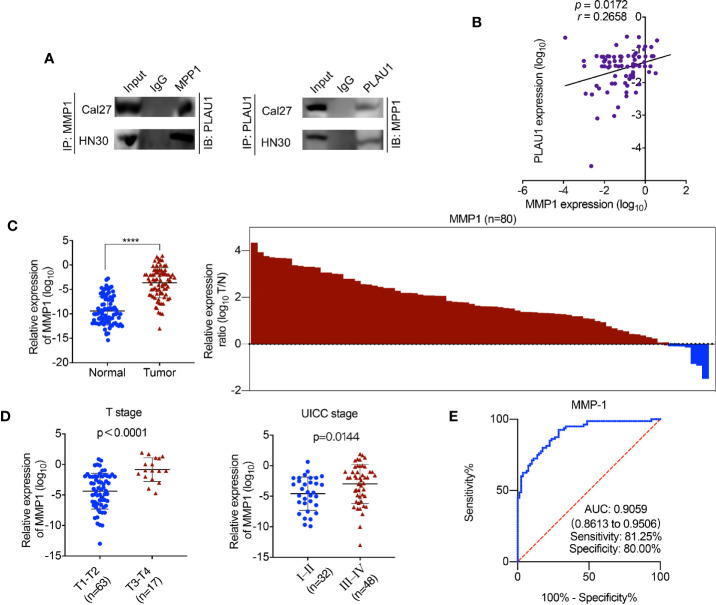
PLAU1 binds with MMP1 in HNSCC. **(A)** The interaction between PLAU1 and MMP1 was determined by co-immunoprecipitation assay. **(B)** A remarkably positive association between PLAU1 expression and MMP1 expression was found using expression correlation analysis. **(C)** The level of MMP1 expression was significantly upregulated in HNSCC tissues (****p < 0.0001). **(D)** Upregulated MMP1 levels were correlated with increased tumor size and advanced tumor stage. **(E)** ROC curves of MMP1 for discriminating HNSCC tissues from adjacent normal tissues.

In our study, the data revealed that the expression level of the MMP1 protein correspondingly increased or reduced following PLAU1 overexpression or knockdown. These results implied that PLAU1 may interact and bind with MMP1 in HNSCC cells. The endogenous interaction between PLAU1 and MMP1 was confirmed by the Co-IP assay (**Figure 4A**). Further analysis revealed that the PLAU1 expression levels were correlated with the MMP1 expression levels (r = 0.2658, p = 0.0172) in HNSCC tissues. (**Figure 4B**). Moreover, a positive correlation between PLAU1 and MMP1 was also observed in the HNSCC samples from the TCGA database (**Supplementary Figure 1C**). In addition, MMP1 expression was also significantly upregulated in HNSCC tissues (**Figure 4C**). An analysis of samples from the TCGA database further revealed that the MMP1 expression was also significantly elevated in HNSCC samples (**Supplementary Figure 1D**). Further analysis revealed that MMP1 was correlated with T stage and UICC stage (**Figure 4D**). The AUC of MMP1 was 0.9059 (95% CI, 0.8613–0.9506; sensitivity: 81.25%; specificity: 80.00%) (**Figure 4E**). Together, these data revealed that PLAU1 interacts with MMP1 and regulates its expression in HNSCC.

### Ectopic Expression of PLAU1 Facilitated Metastasis in Nude Mice

**Figure 5:**
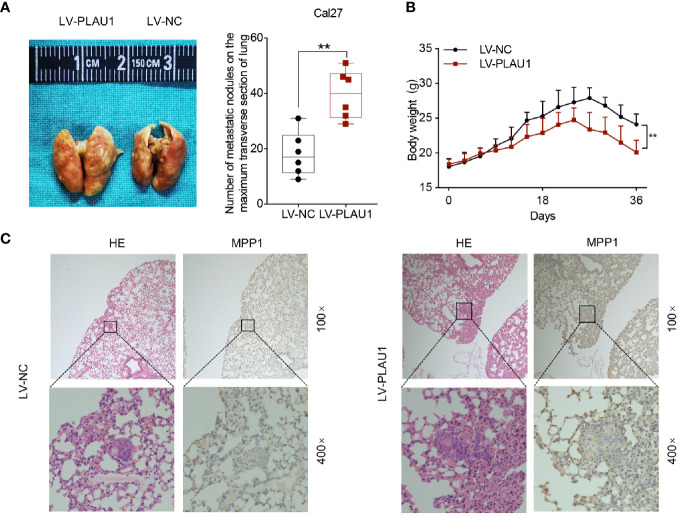
PLAU1 promoted HNSCC cell metastasis *in vivo*. **(A)** Representative images of the lungs of mice injected with Cal27 cells stably overexpressing LV-PLAU1 or LV-NC *via* tail vein injection; the number of metastatic nodules on the maximum transverse section of the lungs was counted (**p < 0.01). **(B)** The body weights of tumor-bearing nude mice injected with Cal27 cells stably overexpressing LV-PLAU1 or LV-NC are shown (n = 6 each group, **p < 0.01). **(C)** Hematoxylin & eosin staining and IHC analysis of MMP1 expression in lung tissues with tumor colonization (the upper row indicates original magnification, 100×; the lower row indicates original magnification, 400×). IHC: Immunohistochemistry.

Since PLAU1 overexpression promotes the malignant properties of HNSCC cells *in vitro*, we also assessed the effects of PLAU1 on tumorigenicity *in vivo*. For this purpose, Cal27 cells consistently overexpressing LV-PLAU1 or LV-NC were injected into nude mice through the tail vein. After six weeks, the mice were killed, and the metastatic nodules of the lungs were detected by picric acid staining. The findings showed that the number of metastatic nodules was dramatically increased in the mice from the PLAU1-overexpressing group compared to those from the control group (**Figure 5A**). The body weights of the mice from the PLAU1-overexpressing group were significantly reduced, compared to those of the mice from the control group (**Figure 5B**). Furthermore, the xenograft tumors in the lungs of mice with PLAU1 overexpression possessed a significantly increased MMP1-labeling index (**Figure 5C**). These results demonstrated that PLAU1 also promoted HNSCC cell metastasis *in vivo*.

### Knockdown of MMP1 Attenuated the PLAU1-Induced Aggressive Effects in HNSCC Cells

**Figure 6:**
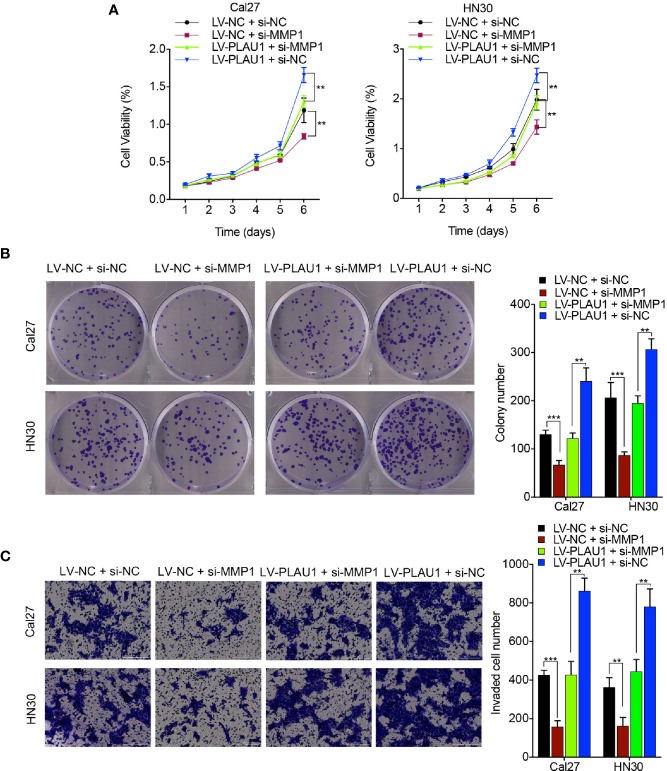
si-MMP1 attenuated the PLAU1 overexpression-induced malignant behaviors in HNSCC cells. **(A–C)** The MTT assays **(A)**, colony-formation assays **(B)**, and Transwell assays **(C)** were performed using HNSCC cells transfected with si-MMP1 or si-MMP1 plus LV-PLAU1. Data are presented as the mean ± SD from three independent experiments (**p < 0.01, ***p < 0.001).

To determine the effects of the coordination between PLAU1 and MMP1 on the biological function of HNSCC cells, the MTT, colony-formation, and Transwell assays were performed. PLAU1 overexpression significantly enhanced the proliferation, colony-formation ability, and invasion ability of Cal27 and HN30 cells, while siRNAs against MMP1 (5’-CCCTAGAACTGTGAAGCATATCG-3’) partially inhibited PLAU1 overexpression-induced cell proliferation (**Figure 6A**), colony-formation ability (**Figure 6B**), and invasion ability (**Figure 6C**), suggesting that PLAU1 affects the proliferation, colony-formation ability, and invasion ability of HNSCC cells *via* interaction with MMP1.

## Discussion

In the present study, the results suggested that PLAU1 was upregulated and associated with poor prognosis in HNSCC. By determining the biological function of PLAU1, we found that PLAU1 enhanced the growth, the colony-formation, migration, and invasion abilities, and the xenograft tumor growth of HNSCC cells *in vivo*. In further experiments, our findings showed that PLAU1 facilitated HNSCC cell proliferation, invasion, and metastasis *via* interaction with MMP1.

Accumulating evidence shows that dysregulation of PLAU1 play an important role in the development and progression of various types of cancers (7–9). A previous study demonstrated that PLAU1 expression levels were significantly decreased in prostate cancers (17). In the present study, our findings revealed that PLAU1 expression was upregulated in HNSCC tissues compared to that in normal tissues and was associated with poor prognosis. The inconsistent PLAU1 expression in malignant tumors may be explained by differences in cancer types. Lin et al. showed that miR-193a-3p suppressed colorectal cancer cell proliferation and migration by regulating PLAU1 expression (18), while another study suggested that PLAU1 participates in the chemoresistance of bladder cancer (8). In the present study, our findings indicated that PLAU1 facilitates HNSCC cell growth and metastasis in both the *in vivo* and *in vitro* experiments. Moreover, PLAU1 has also been identified as a biomarker for predicting overall survival in HNSCC patients (6), and correlated with aggressiveness in HNSCC patients (19). Our results demonstrated that the AUC of PLAU1 was 0.8328 (95% CI, 0.7677–0.8979; sensitivity: 76.25%; specificity: 80.00%) and PLAU1 was correlated with metastatic lymph nodes, T stage and UICC stage HNSCC patients, while the AUC of MMP1 was 0.9059 (95% CI, 0.8613–0.9506; sensitivity: 81.25%; specificity: 80.00%) and MMP1 was correlated with T stage and UICC stage in HNSCC patients. These data indicate that PLAU1 and MMP1 are identified as biomarkers for accurately predicting the prognosis and performing the diagnosis of HNSCC; thus, they may serve as potential targets for clinical therapies to treat HNSCC.

Matrix metalloproteinases (MMPs) are a family of proteolytic enzymes that promote malignant tumor invasion and metastasis by degrading the components of the extracellular matrix (20). Previous studies have shown that MMP1 is upregulated in HNSCC tissues and is associated with poor outcomes (21, 22). A study by Michal revealed that MMP1 could enhance the metastatic ability of HNSCC cells (23), which indicated that MMP1 participates in the development and progression of HNSCC. A previous study showed that PLAU1, MMP1, and MMP2 are involved in signaling pathways related to invasion in breast cancer (24). PLAU1 and MMP1 are associated with the extracellular matrix, cell-cell adhesion, and collagen catabolism in oral squamous carcinoma (25). PLAU1 and MMP1 play similar roles in malignant tumors and exhibit a high degree of connectivity (26). Moreover, PLAU1 was shown to promote disease progression through the secretion of matrix metalloproteinases (MMPs) (27), leading to localized matrix proteolysis (28). A recent study demonstrated that PLAU1 knockdown reduced the migration and invasion abilities of cervical cancer cells by downregulating MMP2 expression (29), which confirmed that PLAU1 influences the biological activities of malignant tumors *via* the regulation of MMPs. Our results showed that PLAU1 interacted with MMP1, and that MMP1 knockdown attenuated the PLAU1-driven aggressive effects in HNSCC cells.

## Conclusion

In summary, our results demonstrate that PLAU1 functions as an oncogenic driver to regulate MMP1 expression in HNSCC. Our findings reveal that PLAU1 may represent a potential therapeutic target for HNSCC.

## Data Availability Statement

The raw data supporting the conclusions of this article will be made available by the authors, without undue reservation.

## Ethics Statement

The studies involving human participants were reviewed and approved by Ethics Committee of the Second Xiangya Hospital. The patients/participants provided their written informed consent to participate in this study. The animal study was reviewed and approved by Ethics Committee of the Second Xiangya Hospital.

## Author Contributions

KW and HW designed this study. KW, Y-YM, N-NH, and SZ performed the experiments. KW and Y-YM wrote the manuscript. All authors contributed to the article and approved the submitted version.

## Conflict of Interest

The authors declare that the research was conducted in the absence of any commercial or financial relationships that could be construed as a potential conflict of interest.
