# Supplementary material for: PLAU1 Facilitated Proliferation, Invasion, and Metastasis via Interaction With MMP1 in Head and Neck Squamous Carcinoma
Source: Front Oncol. 2021 Mar 18;11:574260. doi: 10.3389/fonc.2021.574260 (PMC8013724; doi:10.3389/fonc.2021.574260)
Supplement: Supplementary file 1 [file DataSheet_1.docx]

Supplementary Material

Supplementary table 1. Clinical characteristics of 80 HNSCC patients.

| Group | No. Of patients (%) | |  |
| --- | --- | --- | --- |
| Age(y)  ≥ 60  < 60  Gender  Man  Woman  Primary tumor site  Tongue  Gingiva  Buccal mucosa  Mouth of floor  Hard palate  Oropharynx  UICC stage  I-Ⅱ  III- IV  Perineural invasion  Yes  No  T classification  T1-T2  T3-T4  N classification  Positive  Negative  Tumor grade  Well differentiated  Moderately differentiated  Poorly differentiated  Alcohol history  Yes  No  Smoking history  Yes  No  Adjuvant radiotherapy and chemotherapy  Yes  No | | 49 (61.25)  31 (38.75)  55 (68.75)  25 (31.25)  26 (32.5)  19 (23.75)  17 (21.25)  5 (6.25)  4 (5)  9 (11.25)  32 (40)  48 (60)  20 (25)  60 (75)  63 (78.75)  17 (21.25)  43 (53.75)  27 (46.25)  40(50)  25 (31.25)  15 (18.75)  19 (23.75)  61 (76.25)  23 (28.75)  57 (71.25)  47 (58.75)  33 (41.25) |  |

Supplementary table 2. Sequence of qRT-PCR

| Primers | | Forward | | Reverse |
| --- | --- | --- | --- | --- |
| PLAU1  MMP1  β-actin | GTCGTGAGCGACTCCAAAGGCA  GGTGTCTCACAGCTTCCCAGCG  CCTGGCACCCAGCACAAT | | TTCACAGTGCTGCCCTCCGAA  TCCCGATGATCTCCCCTGACAAAAG  GGGCCGGACTCGTCATACT | |

**
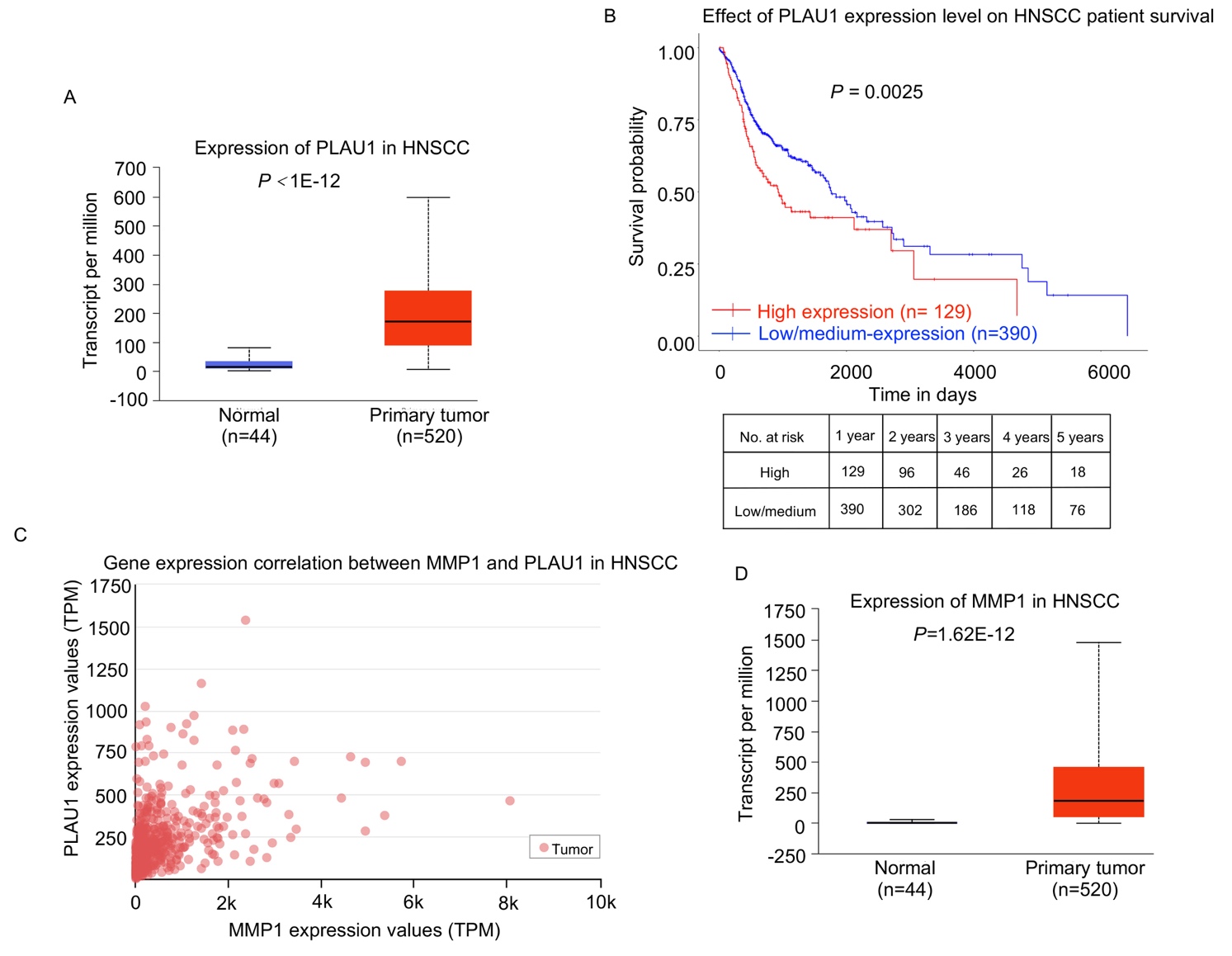
**

**Supplementary figure 1.** The correlation analysis of PLAU1 and MMP1 from TCGA database (A) The level of PLAU1 expression was significantly upregulated in HNSCC tissues (B) PLAU1 was associated with poor prognosis (C) A positive association between PALU1 expression and MMP1 expression was found by expression correlation analysis (D) The level of MMP1 expression was significantly upregulated in HNSCC tissues


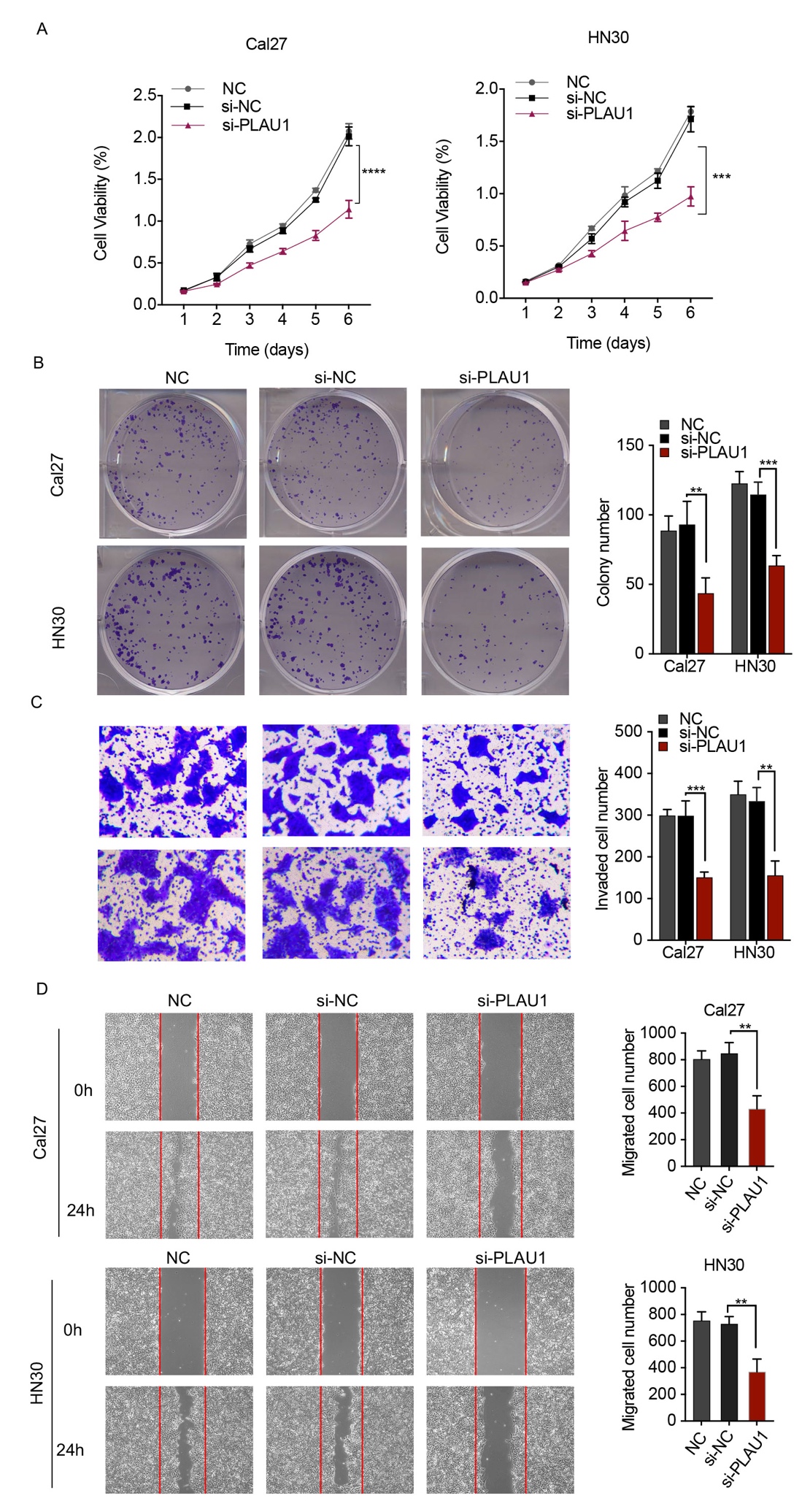


**Supplementary figure 2.** PLAU1 knockdown inhibited HNSCC cell proliferation, migration, and invasion *in vitro*. (A–D) The growth curve and colony-formation, invasion, and migration abilities of HNSCC cells with PLAU1 knockdown were detected using MTT assays (A), colony-formation assays (B), Transwell assays (C), and wound-healing assays (D), respectively.

Data are presented as the mean ± SD from three independent experiments (*p < 0.05, **p < 0.01, ***p < 0.001, ****p < 0.0001). HNSCC: Head and neck squamous cell carcinoma; NC: untreated cell.
